# 

**Published:** 1891-11-28

**Authors:** 


**Notices of Births, Deaths, and Marriages**, are inserted in this column at a charge of 2s. 6d. for an announcement not exceeding four lines.

### NOTICE TO CORRESPONDENTS.

All MS., Letters, Books for Review, and other matters intended for the Editor should be addressed The Editor, The Lodge, Porchester Square, London, W.

The Editor cannot undertake to return rejected M.S., even when accompanied by stamped directed envelope.

**Notice to Advertisers and Subscribers.**

All Advertisements, Orders for Copies of The Hospital, and Business Communications should be addressed to The Publisher, *not to the Editor*, The Hospital, 140, Strand, W.C.

The Cost of Subscription, post free, is as follows:—Per week, 1½d.; per quarter, 1s. 8d.; per half-year, 3s. 3d.; per annum, 6s. 6d. Foreign:—Per annum, 8s. 8d.; payable, in all cases, in advance.

**"Burdett's Hospital Annual," 1891—1892.**

Third year of publication. 600 pp. Boards, gilt lettering. A most complete guide to British and Colonial Hospitals, Dispensaries, Nursing and Convalescent Institutions, and Asylums. Price 3s. 6d. Published by The Hospital (Limited), 140 Strand, W.C.

**NOTICE.—The Index for Vol. X. (ending September, 1891), is on sale at the Office of this Journal, price 2½d., post free.**

### General Charities.

[This Column is devoted to an account of work of charitable institutions other than Medical Charities. The Editor will be glad to receive reports or news of work at 140, Strand. *Philanthropy* should be plainly written on the envelope.]

The activity of the Society for the Prevention of Cruelty to Children is shown by the fact that during the last quarter 2,181 complaints of cruelty, involving the welfare of 5,128 children, were investigated. Neglect and starvation head the list of classified cases by a large majority.

The Rev. R. Milburn Blakiston appeals for fresh contributions to the **Incorporated Church Building Society**, 7, Dean's Yard, S.W. He writes, that "at the last meeting of the Committee all available funds were voted in grants to churches and mission buildings. Since October, 1890, £3,740 have been voted towards 74 churches, and £690 towards 27 mission buildings."

In consequence of the Sunday cargo-working ordinance having come into operation, a remarkable diminution is announced by the **Missions to Seamen Society** to have taken place in the number of steamers working cargoes on Sundays in Hong Kong harbour. In April last as many as 68 steamers were so working cargoes, whereas in August the number had fallen to four. This relaxation is naturally a great boon to mercantile marine officers, and as in the long run it is not likely to prove detrimental to the general interests of trade, the Society looks forward hopefully to seeing similar restrictions on Sunday labour established ere long in the ports of our Indian possessions.

The Hon. Treasurer of Miss Charlotte Mason's **House of Rest** issues an appeal dated from the London and South-Western Bank, Kilburn, for the sum of £1,500 to pay off the amount advanced to Miss Mason to enable her to complete the purchase of the new premises to which the institution was recently removed. Miss Mason, it appears, is anxious to add a wing to the building to be called the Aged Workers' Home, in which separate rooms shall be provided, rent free, with firing, for those aged women who have devoted their lives to missionary or philanthropic work. This extension, no doubt, will not be begun till the debt to which we have referred has been paid off.

Mr. Francis A. Bevan, who presided at the half-yearly meeting in connection with the **British Home for Incurables**, announced that an appeal is about to be made to the clergy throughout the country for offertories. The explanation of the reason for not electing so many patients on this occasion as usual is to be found in the fact that the finances of the institution did not warrant the election of more than five patients. A more satisfactory announcement from the Chairman was that owing to the generosity of two anonymous friends and some City companies, the management had been enabled to wipe out the debt with which the Institution was burdened. Having got rid of this incumbrance, there seems no reason why the society should not steer a clear course for the future.

Mrs. Bradley appeals for contributions, either of money or clothes, for **The Working Women's House**, 53, Horseferry Road, Westminster. The work is carried on on the principle that "Prevention is better than Cure," and the House is a combination of a lodging-house for young servants and other women of good character, who are in search of employment, a registry for respectable charwomen, and a workroom. She pleads more particularly for the department for training girls of the age of 13 or 14, who are selected from poor homes and brought to the association, in order to be taught the elements of cooking, washing, and house-work generally, and be prepared for domestic service. As Mrs. Bradley points out, the only chance of giving many poor children any notion of obedience, order, cleanliness, and truthfulness, is to begin their training as soon as possible after leaving school. Far too many children have joined the ranks of the criminal classes from not having been taken in hand when still young and impressionable for good.

The announcement of the Postmaster-General that in future his department will give employment as postmen to time-expired soldiers of good character, who pass the necessary educational standard, is earnest of an endeavour by the State to do what **The Association for the Employment of Soldiers** has been doing unostentatiously for the past six years. The Government has contributed annually the munificent sum of £200 towards the institution. Such a contribution cannot be called an extravagant one; in fact, governments, as every properly-informed citizen well knows, never are guilty of extravagance in any direction, except in the minds of those people who are so unkind as to hint at money mis-spent on an army that cannot march and ships which cannot float. Unfortunately, the association, which now has 26 branch offices in some of the principal towns of the kingdom, is much crippled for want of funds. During the six years of its existence it has found work for some 7,000 men; during last year alone it obtained situations for over 2,000, representing in the aggregate wages amounting to nearly half a million. There is, however, scope for much more exertion, with ampler funds more branches could be opened. No fee is charged either to men or employers, and the employments for which men are best suited are those of servants, clerks, musicians, and railway porters. The fact that they have been taught habits of regularity and discipline should make them useful servants. Those who are willing to support the association should send donations to the central office, 12, Buckingham Street, Strand.

### Scraps and Gleanings.

Hertford and Ware have come to an agreement to erect a joint infectious diseases hospital.

Cork holds its first Hospital Saturday Collection on November 28th. It is intended to be pre-eminently a working man's collection.

The Croydon General Hospital presents a very favourable report this year, and an extension of the accommodation will be probably necessary before very long.

The death of William John M'Cullough, of lockjaw, is reported, his thumb having been broken during a quarrel with his father, Edward M'Cullough.

The new Fever Hospital at Burton boasts of being one of the best temporary hospitals in the country. It was designed by the Borough Surveyor, Mr. Swindlehurst.

The Bishop of Oxford opened a bazaar at Reading, under the patronage of Princess Christian of Schleswig-Holstein, in aid of the Oxford Diocesan Branch of the Ladies' Home Mission Association.

The Local Board of Enfield have agreed that "No small-pox patients shall be received into the temporary hospital about to be erected," and that the buildings shall be entirely removed at the end of two years.

Princess Louise Marchioness of Lorne opened last week the new buildings, and distributed the prizes to the pupils of the Sydenham High School of the Girls' Public Day School Company, of which her Royal Highness is patroness.

A luncheon was given at the Woodlands Convalescent Home, Bradford, to celebrate the transfer of the establishment from the hands of the present administration to the management of the Bradford Infirmary. It is thus practically a free gift to the town of Bradford by the late Sir Edward Ripley.

The mail steamship "Alliance," on arriving at New York, reported that yellow fever was raging at Santos, Brazil, when she left that port. The surgeon and three of the crew of the "Alliance" died of yellow fever on the voyage. The vessel was allowed to pass by the quarantine authorities, and the passengers landed.

The benefit in aid of the Gordon Boys' Home, at Chobham, will be given at the Royal Aquarium, Westminster, on Thursday, the 26th inst., and two following days. On each day there will be a continuous performance from ten a.m. until half-past eleven p.m., the programme including entertainments by many of the best known artists.

Once set a ball rolling, and no one can say when it will stop. The ball of hospital investigation has unfortunately started on its course, and hardly a week passes without some fresh instance of adverse criticism respecting some particular institution. The latest victim is the Wolverhampton Eye Infirmary. May the charges prove groundless, and silence complaints which do serious injury to useful institutions.

### THE STUDENT OF MEDICINE.

[All communications for this Supplement should be addressed to THE EDITOR of THE HOSPITAL, at 140, Strand, with the words, "Students' Column," written in the left hand top corner of the envelope.]

### EDITORIAL NOTES.

CRICKET.—An interesting lecture on the subject of cricket was recently given, at the Westminster Town Hall, by Mr. Frederick Gale. In this he first gave some of his own reminiscences of the noble game and of its votaries. A brief review or historical sketch of cricket was also given, in which it was pointed out that the germ of the game as now played was to be found among the Greeks and Romans. One of its earliest followers in this country was Oliver Cromwell. For some time after the accession of George the First it remained under a cloud, the Government in those Jacobite days being unwilling to permit a large number of people to collect together, and the *Gentleman's Magazine* subsequently took up the cudgels against it. Some idea of the opposition to the game might be gathered from such works as "Waverley" and "Red Gauntlet." The present game was a sort of mongrel, since it was evolved from rounders, club ball, hockey, tip-cat, and other ancient sports. To illustrate a point in its national character, it was remarked that its laws had been much the same for 120 years, and the wicket the same since 1817. A description of a village cricket match of sixty years ago was then given, of old Lord's about 1841, and of the changes the lecturer had noticed in the style and art of bowling. He mentioned in the course of the lecture that Mr. Richmond, the R.A., now in his 83rd year, had been present at no fewer than seventy-three matches between the Gentlemen and the Players. The cricket-grounds had wonderfully improved in recent times, in fact the lecturer thought sometimes that they had been rendered too easy, in fact, too much like a billiard-table. Some remarks were finally made upon the difference made in the conditions by the introduction of boundary hits.

### THE LONDON HOSPITAL.

AT the meeting of the House Committee on Tuesday, November 24th, 1891, Dr. W. J. Hadley was appointed Medical Registrar of the London Hospital for the ensuing year. Some exceedingly good candidates came forward, and the Hospital School is to be congratulated on the appointment of Dr. Hadley, who held his first resident appointment as House Surgeon in 1884, after having completed his course of study at the school there.

### APPOINTMENTS.

At King's College Hospital, Howard Distin, M.R.C.S., L.R.C.P., has been appointed Sir Joseph Lister's house surgeon; B. W. Longhurst, M.R.C.S., L.R.C.P., Mr. Cheynes' house surgeon; M. K. Soutter, P.R.C.S., L.R.C.P., Mr. Rose's house surgeon; E. L. Pritchard, L.R.C.P., M.R.C.S., assistant house physician; J. J. Waddelow, L.R.C.P., M.R.R.S., L.S.A , house accoucheur; J. H. Dempster, L.R.C.P., M.R.C.S., assistant house accoucheur; and A. Whitefield, L.R.C.P., M.R.C.S., house physician. At the Hospital for Sick Children, Great Ormond Street, Charles Arkle, M.D., M.R.C.P., has been appointed medical registrar and pathologist. At the National Hospital for Epilepsy, Queen Square, H. N. Bournan, M.B., M.R.C.S., L.R.C.P., has been appointed junior house physician, and Henry M. Bowman, M.B., M.R.C.S., L.R.C.P., house physician. At the Charing Cross Hospital, Richard Hawtree Collins, M.R.C.S., L.R.C.P., has been appointed resident obstetrical officer, and John Harrold, L.R.C.P., M.R.C.S., medical registrar. At the Westminster Hospital, E. Lloyd-Williams, M.R.C.S., L.D.S. Eng., L.R.C.P., L.S.A., has been appointed surgeon dentist. At the Huddersfield Infirmary, Robert C. Brown, M.B., B.S. Dun., has been appointed senior assistant house surgeon, and G. Francis Rhodes, M.B., C.M. Edin., senior house surgeon. At Owen's College, Manchester, A. J. Chalmers, M.B., Ch.B., has been appointed junior demonstrator of anatomy. At the Royal Hants County Hospital, Winchester, J. Tertius Clarke, M.R.C.S., L.R.C.P., has been appointed house surgeon. At the New Hospital for Women, Euston Road, Emile Louisa Dove, M.B. Lond., has been appointed resident medical officer. At the East London Hospital for Children, Shadwell, C. B. James, M.R.C.S., L.R.C.P., has been appointed house physician. At the Seamen's Hospital Society,

### The Hospital Advertiser.

### MEDICAL APPOINTMENT.

THE HOSPITAL FOR SICK CHILDREN,

### THE STUDENT OF MEDICINE—(*continued*).

A. Jervis, M.R.C.S., L.R.C.P., has been appointed house surgeon. At the Hastings, St. Leonards, and East Sussex Hospital, William Henry Spencer, M.A., M.D. Camb., M.R.C.P. Lond., has been appointed honorary physician.

### VACANCIES.

The following vacancies are announced this week, the amount of salary in each case being given after the name of the institution:

**Resident Medical Officers.**

City of London Hospital for Diseases of the Chest, House Physician—Board, &c.
Darlington Hospital and Dispensary, House Surgeon—Salary £100, with board, &c.
Dudley Dispensary, Resident Medical Officer—Salary £130.
North-Eastern Hospital, for Children, Junior House Surgeon—Salary £60.
Northern Infirmary, Inverness, House Surgeon and Apothecary—Salary £100, with board.
North Staffordshire Infirmary and Eye Hospital, Hartshill, Stoke-upon-Trent, House Physician—Salary £100, with board, &c.; also Assistant House Surgeon—Board, &c.
Royal Hospital for Diseases of the Chest, City Road, House Physician—Salary £40, with board, &c.; also Assistant House Physician—Board, &c.
Victoria Hospital for Sick Children, Hull, House Surgeon—Salary £50, with board.
West London Hospital, Hammersmith Road, House Physician; also House Surgeon—Board, &c.

**Non-Resident Medical Officers.**

Chelsea, Belgrave, and Brompton Dispensary, Physician.
City of London Hospital for Diseases of the Chest, Pathologist—Salary £105.
Dental Hospital of London, Leicester Square, Dental Surgeon and Assistant Dental Surgeon.
Great Northern Central Hospital, Dental Surgeon.
Hospital for Sick Children, Great Ormond Street, Surgical Registrar—Honorarium £40.
Owens College, Manchester, Professor of Pathology—Salary £650.
Royal Hospital for Diseases of the Chest, Assistant Physician.

### NOTES FROM THE SCHOOLS.

**Edinburgh University.**

Mr. Goschen paid a visit to Edinburgh for the purpose of delivering the customary address, as being the Lord Rector of the University. Mr. Goschen arrived at Edinburgh on the 18th, and was met at the station by Sir William Muir (Principal of the University), Lord Stourmouth Darling (the Lord Rector's Assessor on the University Court), and Mr. L. C. D. Douglas (President of the Students' Representative Council). In spite of the heavy rain and the late hour (eleven o'clock), a torch-bearing procession of about 500 students met Mr. Goschen's carriage as it drove out of the station. The procession had previously formed in the Old University quadrangle, and had an escort of about a dozen mounted police, while each side had a row of police running the whole length of the procession. Many of the students were in fancy dress, one being a "Mahatma." The University Hall was lighted up, and several rockets were let off as the Lord Rector passed. The procession escorted Mr. Goschen to Lord Kyllachy's house, whose guest he was during his stay here. After Mr. Goschen had made a brief speech, the procession passed on to the castle-yard, where a bonfire was made of the torches. After that the students' quarters was made for. The way home was enlivened by songs, although, to the credit of those in the procession, the Royal Infirmary was passed in silence. The mounted police here attempted to disperse the procession, but were unsuccessful. Next day Mr. Goschen delivered his address in the music hall. The building was crowded with students long before the proceedings began, the time being filled up by the singing of students' songs—"John Peel," "Clementine," "The Tarpaulin Jacket," "Whelks and Mussels," formed the official programme, to which Mr. John Greigs, Mus. Doc., played the organ accompaniment. On the platform were all the university officials, and numbers of the other academic and municipal dignitaries of Edinburgh. The Rev. Professor Taylor, D.D., Dean of the Faculty of Divinity, having opened the proceedings with prayer, Professor Kirkpatrick, LL.D., Dean of Faculty of Law, presented Mr. Goschen for the degree of Honorary LL.D., which was conferred on him by the Vice-Chancellor. The chairman, Sir William Muir, then called on Mr. Goschen, who was received with great applause, to deliver his address. He took for his subject "The Faculty of Imagination," and worked out its importance in all branches of literature and science; from poetry, romance, to mathematics, and political economy. His address was admirably suited in every way to his audience, consisting, as it did, of students of medicine, law, arts, and divinity. A vote of thanks to Mr. Goschen was proposed by Mr. L. C. D. Douglas, President of the

THE STUDENT OF MEDICINE—(*continued*).

Students' Representative Council, and one to the Chairman by Mr. A. Stodart Walker, M.B., C.M., President of the Union. After this meeting was over Mr. Goschen paid a visit to the University Union, being accompanied by Sir William Muir, and was received by the President and Committee. Mr. Goschen, in the debating hall made a short speech to the members present, saying that it (the Union) had a special interest to him, as he had been, when at Oxford, President, Secretary, and Treasurer of that University's Union, but this Union exceeded considerably in architectural beauty and comfort the Oxford one, and he believed also that of Cambridge. He congratulated the members on their possession. In the evening Mr. Goschen presided at a University Smoking Concert for which a thousand tickets were issued. Almost the entire profession were present. The band of the Cameron Highlanders performed. Among the vocalists was Professor Rutherford.

On November 20th Mr. Henry Irving visited the University Union and made a short speech to the members present. Edinburgh had a special attraction for him, as it was here that he first studied his profession. In reference to his being an Honorary President of the Union Dramatic Society he made this offer, that if the Dramatic Society would perform the Merchant of Venice he would take Shylock's part, and Miss Terry would be Portia. This extremely kind offer was received with great applause. Three cheers were given for Mr. Irving and Miss Terry.

### STUDENTS' MEDICAL SOCIETIES.

### Guthrie Society, Westminster Hospital.

The monthly meeting of the above society came off on Thursday, November 12th. Dr. Allchin, the President, was in the chair. After hearing pro and con. arguments, it was decided to hold debates on certain nights of the meetings of the society. After settling the above question, the President called upon Mr. Nitch-Smith to read his paper entitled "A Short Outline of the History, Climate, &c., of the Cape Colony—Cape of Good Hope." The paper proved most interesting, and if the amount of applause and the reception of the reader may be taken as a measurement of praise, Mr. Nitch-Smith can be congratulated on what he called his first appearance before a public gathering.

### ATHLETICS.

### Football.—Rugby, November 10th.

*Edinburgh University v. Bradford.*—This biennial match was played at Raeburn Place, Edinburgh. The 'Varsity team was overmatched in skill and in training by the Yorkshiremen. The game, which was exciting throughout, ended in a victory for Bradford by three goals and a try to nil.

November 14th.

*University College Hospital* was beaten by *Brockley* by two goals and two tries to nil. *Guy's Hospital* was beaten by *Croydon* by a goal from a try by Bacon, to a goal kicked from a fair catch. *St. Bartholomew's Hospital* was beaten by R. I. E. C. This match which was played before a numerous gathering of visitors at Cooper's Hill, resulted in a victory for the home team by a dropped goal by Sangster, and six tries to nil. *London Hospital* drew with *Sidcup*, at Sidcup, neither side scoring. *Middlesex Hospital* beat Wickham Park, at Lee, by a goal and three tries to nothing. *Westminster Hospital* beat *Christ's Hospital* at Penge, by four tries to one goal and one try.

Association, November 14th.

*St. Thomas's Hospital* was beaten by the *Forest School* at Wanstead by six goals to three. *Bartholomew's Hospital* was beaten by *London Welsh* by a goal to nil. *St. Mary's Hospital* beat *Old Salways*. at Wimbledon, by three goals to one. *Bart's* drew with *Olympian* at Walthamstow, each side scoring four goals.

Rugby, November 21st.

*Middlesex Hospital* was beaten by *Clapham Park*, at Balham, by six points to nil. *St. Bartholomew's Hospital* was beaten by *Croydon*, at Croydon, by a goal and two tries to nil. *Guy's Hospital* 2nd beat *Kensington* 2nd, at Raynes Park by a goal to a try. *Middlesex Hospital* beat *Lennox*, at Dulwich, by a try to nothing.

*St. Thomas's Hospital v. London Scottish.*—This match was played at Richmond in very dull weather, but before a good company of spectators. Both sides were well represented, and the match resulted in a very good game. At half time one try each had been scored. In the second half the Scotchmen added another try to their score, and thus a capital game was won by the Scotchmen by two tries to one, or four points to two.

*Dental Hospital* drew with *Pendragon*, at Clapham, neither side scoring. *University College Hospital* bea *Sutton*, at Sutton, by a try to nil. *London Hospital* was beaten by *Saracens*, after a closely contested game, by a goal from a try by Swyer, who converted it to a try by Mortimer.

Association, November 21st.

*King's College Hospital* was beaten by *Old Salways*, at Leyton, by four goals to one. *St. Bartholomew's Hospital* was beaten by *Royal Arsenal* by nine goals to none. *St. Thomas's Hospital* beat *Surbiton Hill* by two goals to nil.

